# Prevalence and outcomes of multimorbidity in South Asia: a systematic review

**DOI:** 10.1136/bmjopen-2014-007235

**Published:** 2015-10-07

**Authors:** Sanghamitra Pati, Subhashisa Swain, Mohammad Akhtar Hussain, Marjan van den Akker, Job Metsemakers, J André Knottnerus, Chris Salisbury

**Affiliations:** 1 Indian Institute of Public Health, Bhubaneswar, Public Health Foundation of India, Bhubaneswar, Odisha, India; 2 Division of Epidemiology and Biostatistics, School of Public Health, University of Queensland, Brisbane, Queensland, Australia; 3 Department of Family Medicine, School CAPHRI, Maastricht University, Maastricht, The Netherlands; 4 Department of General Practice, KU Leuven, Leuven, Belgium; 5 Centre for Academic Primary Care, School of Social and Community Medicine, University of Bristol, Bristol, UK

**Keywords:** PUBLIC HEALTH, PRIMARY CARE

## Abstract

**Objective:**

To systematically review the studies of prevalence, patterns and consequences of multimorbidity reported from South Asia.

**Design:**

Systematic review.

**Setting:**

South Asia.

**Data sources:**

Articles were retrieved from two electronic databases (PubMed and Embase) and from the relevant references lists. Methodical data extraction according to Preferred Reporting Items for Systematic reviews and Meta-Analyses (PRISMA) guidelines was followed. English-language studies published between 2000 and March 2015 were included.

**Eligibility criteria:**

Studies addressing prevalence, consequences and patterns of multimorbidity in South Asia. Articles documenting presence of two or more chronic conditions were included in the review. The quality and risk of bias were assessed using STROBE criteria.

**Data selection:**

Two reviewers independently assessed studies for eligibility, extracted data and assessed study quality. Due to heterogeneity in methodologies among reported studies, only narrative synthesis of the results was carried out.

**Results:**

Of 11 132, 61 abstracts were selected and 13 were included for final data synthesis. The number of health conditions analysed per study varied from 7 to 22, with prevalence of multimorbidity from 4.5% to 83%. The leading chronic conditions were hypertension, arthritis, diabetes, cardiac problems and skin diseases. The most frequently reported outcomes were increased healthcare utilisation, lowered physical functioning and quality of life, and psychological distress.

**Conclusions:**

Our study, a comprehensive mapping of multimorbidity research in South Asia, reveals the insufficient volume of work carried out in this domain. The published studies are inadequate to provide an indication of the magnitude of multimorbidity in these countries. Research into clinical and epidemiological aspects of multimorbidity is warranted to build up scientific evidence in this geographic region. The wide heterogeneity observed in the present review calls for greater methodological rigour while conducting these epidemiological studies.

**Trial registration number:**

CRD42013005456.

**Strengths and limitations of this study**

- Our systematic review identifies a large knowledge gap in the epidemiology of multimorbidity in South Asia, where few studies have been conducted to investigate multimorbidity.
- Our review is the first to undertake a comprehensive mapping of multimorbidity studies in South Asia and demonstrates the need for systematic enquiries on multimorbidity to be undertaken in this region.
- Since multimorbidity is not well indexed in literature databases, we might have inadvertently omitted some studies.
- A quantitative synthesis could not be performed due to a large amount of heterogeneity among the selected studies.

## Introduction

In the past few decades, chronic diseases have replaced infectious diseases and assumed the dominant healthcare burden.^1^ Coexistence of multiple chronic diseases in a single individual, known as multimorbidity, is increasingly becoming the norm.^2^ Individuals with multimorbidity register higher mortality rates, incur increased healthcare expenditure, are frequently hospitalised, and experience disturbed physical and mental health, affecting overall functioning and quality of life.^3^ ^4^ Owing to its negative consequences and high resource use associated, multimorbidity has attracted considerable interest and attention among clinicians and public health researchers alike.^5^ A considerable corpus of primary care research over the last decades has been performed around this area, in developed countries.^6–9^ Prevalence estimates in these countries have shown varying figures ranging from 39.5% in Spain to 13% in the Netherlands.^10^ ^11^ A study involving primary care patients in Scotland has revealed one quarter of patients to have multimorbidity, with one-third of them being young.^12^ This study strongly urged the global health community to be adequately prepared to be responsive to the challenges of multimorbidity. Nonetheless, the population-based studies from several middle income countries such as Ghana, Brazil and South Africa reported prevalence of multimorbidity as high as 38.5%.^13–15^ However, to date, the majority of research from low- and middle-income countries (LMICs) are focused on a single or specific illness, or on the coexistence of a relatively small number of diseases such as cardiovascular ailments, diabetes and cancer, and the presence of unrelated or incongruent multiple chronic conditions has not been investigated in detail.^16^ ^17^

Together, home to approximately one-fifth of the world's population, South Asia deserves special attention in the context of multimorbidity. All of the seven countries in this region are LMICs.^18^ Along with rapid urbanisation and demographic transition, these countries are now experiencing a shift from communicable to non-communicable diseases, and multimorbidity could be an emergent phenomenon. Given the high younger population, the projected magnitude can be enormous, and the extant unprepared health system and limited resources could cumulatively add to the adverse impacts.

Several studies are available from individual South Asian countries on the level of selected or individual chronic diseases among the adult population. However, to the best of our knowledge, to date, there are no comprehensive systematic reviews on multimorbidity among adults residing in the South Asian region, and therefore a contextual understanding essential for developing and aligning health services to meet patient care is lacking. The present systematic review is the first attempt to landscape multimorbidity research in South Asia and to systematically evaluate published studies (longitudinal, cross sectional) documenting occurrence, pattern and consequences of multimorbidity in the adult population in South Asian countries, thus enabling comparison with other regions. It is expected that the information acquired would identify existing knowledge gaps and guide future research needs into multimorbidity in this region. The specific objectives were to (1) estimate the prevalence of multimorbidity, and (2) study the patterns of occurrence and its consequences in South Asia. The focus of the review was limited to multimorbidity defined as the co-occurrence of multiple chronic diseases in the same individual or mean disease count per individual.

## Methods

A systematic review of published studies reporting multimorbidity among adults residing in South Asia was undertaken in accordance with the Preferred Reporting Items for Systematic reviews and Meta-Analyses (PRISMA) statement. The methodology has been published in PROSPERO with registration ID: CRD42013005456. (http://www.crd.york.ac.uk/PROSPERO/).

### Inclusion and exclusion criteria

Selection of articles was based on following inclusion criteria, they were: (1) original studies documenting prevalence, patient factors associated with multimorbidity and consequences of it; or (2) studies reporting results that allowed calculation of prevalence; (3) studies having participants of more than 18 years of age; (4) conducted either in a primary care/outpatient setting or general population from the above mentioned South Asian countries; (5) studies that had published results between 1990 and March 2015. As multimorbidity first came to prominence in the early 1990s, we included articles published in the English language between 1 January 1990 and 31 March 2015.

For those studies in which multimorbidity was not defined, we made an operational definition of ‘studies documenting two or more chronic conditions, even though not mentioning the term multimorbidity’. These were also included for data synthesis. Any study that began with a preliminary selection of index disease (studies of comorbidity) was excluded.

### Search strategy for identification of articles

We systematically explored Pub Med and EMBASE electronic databases, and Google Scholar search engines, to locate the relevant articles. We categorised the search terms according to location, methodology and outcomes: (1) Location: ‘India, Pakistan, Nepal, Bhutan, Bangladesh, Sri Lanka, Maldives, South Asia’. 18 (2) Method: ‘prevalence, epidemiology, cluster, pattern’. (3) Outcome: ‘multimorbidity, multimorbid, multi-morbidity, multiple conditions, co-morbid, multiple diseases, multiple chronic diseases, multiple chronic conditions, multiple illnesses, multiple diagnoses, multi-pathology, chronic condition, chronic diseases’. The ‘AND’ Boolean operator was used to combine search terms across the categories and ‘OR’ was used to combine within the categories. To broaden the scope of our research, we also applied the linguistic variations of multimorbidity in the search strategy. Further, we limited the search to those studies that only involved human participants, had abstracts available and were published between 1 January 1990 and 31 March 2015. To obtain additional publications, reference lists of retrieved articles were hand searched using snowballing techniques. Wherever possible, forward citations of the studies retrieved during the literature search were traced and screened for possible inclusion. Furthermore, search of relevant websites, namely multimorbidity research network of university of Sherbrook (http://crmcspl-blog.recherche.usherbrooke.ca/) and WHO (http://www.who.int/en/), was also performed. A summary of the search strategy adopted for the review is outlined (see online supplementary appendix 1).

### Data management

First, all hits obtained were gathered and duplicates removed. Potentially relevant articles were selected through initial title and abstract screening by two authors (MAH and SS) independently. In the next step, the full text copies of these relevant articles were retrieved. We retained those articles that studied the prevalence of more than two chronic conditions without any index disease, even if they were not using the term ‘multimorbidity’. Articles meeting all inclusion criteria were retained for quality assessment and data extraction. For data extraction, a special form was constructed. Two authors (MAH and SS) independently assessed each of these 61 retrieved articles for inclusion, extracted data and cross checked data extraction forms. Any discrepancies regarding eligibility between the two reviewers were resolved by consensus with two other authors (SP, CS).

### Assessment of study quality

Two authors (MAH and SS) independently assessed risk of bias and study quality, using standard ‘strengthening the reporting of observational studies in epidemiology’ (STROBE) checklist.^9^ ^19^ Any disagreement arising on quality was sorted out in consultation with two other authors (SP and CS). For observational study designs, risk of bias was assessed for three domains: selection bias, information bias (differential misclassification and non-differential misclassification) and confounding. Risk of bias for each domain was assessed as either ‘Yes’ or ‘No’. Studies that had a risk of bias in each domain, including a risk of confounding, were classified as having more of a risk of bias. Each reviewer independently determined a global quality score for each article, giving one point for each STROBE item the article addressed. To be retained in our review, articles had to have a quality score of at least 12 of a possible 23.

### Data extraction

For each included study, we extracted the following information: (1) authors and publication year; (2) title and journal; (3) study country and location (urban or rural); (4) study design; (5) sampling strategy (random or non-random); (6) sample size; (7) sample characteristics such as age and gender; (8) number of conditions included; (9) definition of multimorbidity considered; (10) prevalence (overall and gender- or location-specific) of multimorbidity; (11) consequences of multimorbidity in terms of health-related quality of life (HRQoL), functional status, healthcare utilisation and healthcare expenditure (objective or subjective); and (12) risk factors significantly associated with multimorbidity.

We decided not to perform meta-analysis as we judged that the included studies were heterogeneous in different aspects, including: populations (different ages and settings), variable definitions (including different definitions of exposures and outcomes) and analytical strategies (adjustment for different confounders).

## Results

### Yield of search strategy

The searches mentioned above yielded 11 132 articles. After discarding duplicates, 11 021 were selected for title screening. Careful screening of titles identified 189 articles for abstract reading, from which 61 were shortlisted for full text review. Finally, 13 articles were included for this systematic review. Reasons for exclusion of the remaining articles are indicated in figure l.

**Figure 1:**
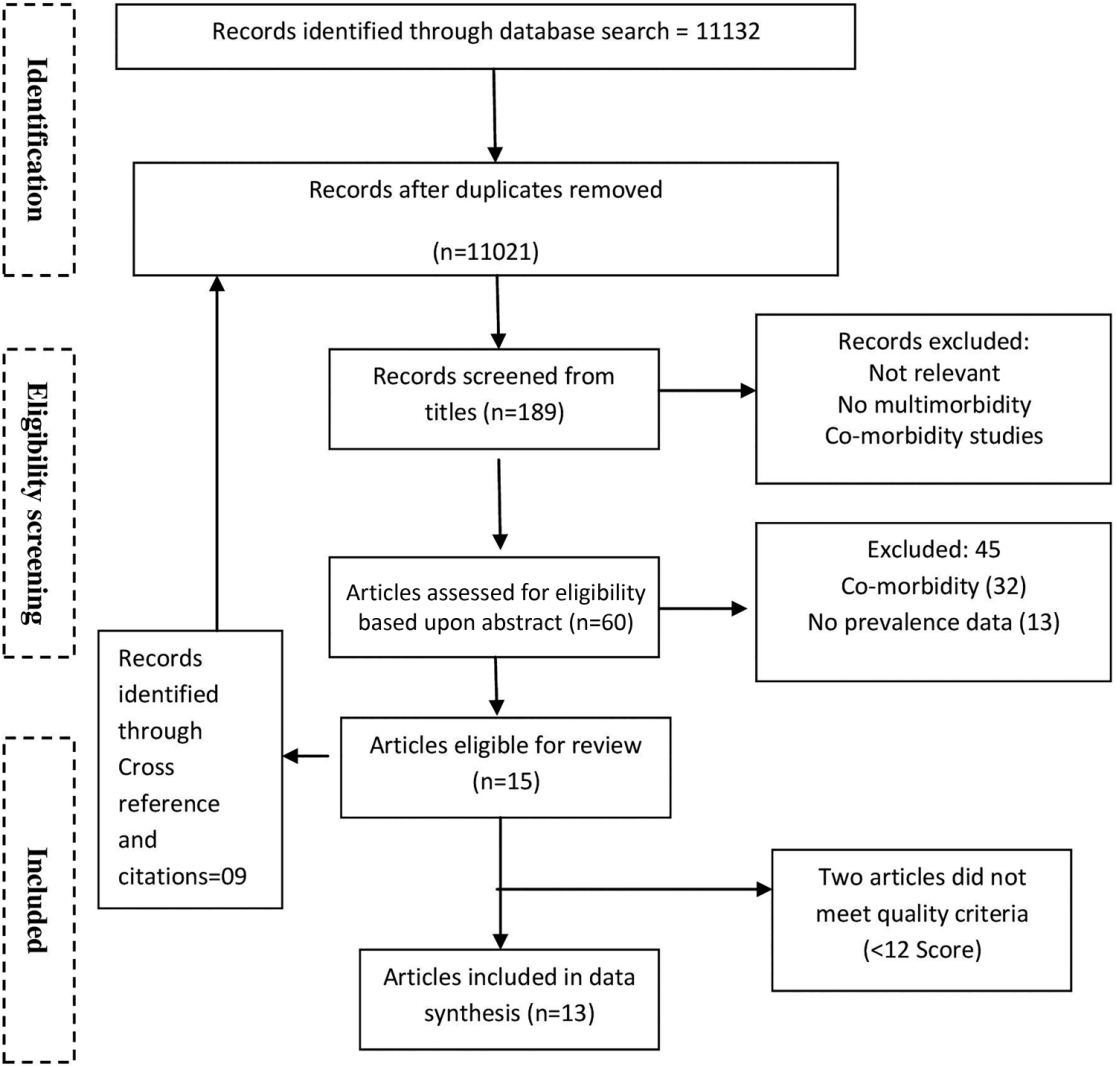
PRISMA (Preferred Reporting Items for Systematic reviews and Meta-Analyses) flowchart of the literature search.

### Study characteristics and quality

The key characteristics of the studies are presented in table 1. All the studies were from India and Bangladesh. Four studies were carried out on a nationally representative sample and the rest adopted ad hoc study designs.^20–23^ All were cross-sectional and quantitative in nature. All studies were community based. Six studies recruited participants exclusively from rural areas,^24–29^ two from urban area^16^ ^30^ and five included urban as well as rural participants.^20–23^ ^31^ The sample size of the included studies varied from 90 to 44 514, and included males as well as females. Seven studies exclusively included participants over 60 years of age.^24–28^ ^30^ ^32^ Proportion estimation was the most frequently used statistical measure. On a quality assessment scale, five studies scored between 12 and 18^24^ ^26^ ^27^ ^29^ ^30^; whereas eight studies scored more than 18,^16^ ^20–23^ ^25^ ^28^ ^32^ and five articles scored between 12 and 18 (see online supplementary appendix 2).

**Table 1:** Studies reporting prevalence of multimorbidity in South Asia

Study Settings				Sample			Methods	
Sl no	Study (year)	Country	Study period	Sample size	Age in years	Settings	Data collection	Number of diseases included
1	Joshi *et al*, 2003^32^	India	July 1999–April 2000	200	>60	Community based (urban and rural)	Self-reported, medical records, Physician’s diagnosis	27, ICD10 coding related to diseases
2	Purty *et al*, 2006^24^	India	October 2002–October 2003	320	>60	Community based (rural)	Self-reported, Physician’s diagnosis, Laboratory investigations	15
3	Khanam *et al*, 2011^25^	Bangladesh	July 2003–March 2004	625	>60	Community based (rural)	Physician's diagnosis, Laboratory investigations	9
4	Chakraborty *et al*, 2004^27^	India	January–March 2005	420	>60	Community based (rural)	Self-reported	>12
5	Swami *et al*, 2002^30^	India	1998–1999	362	>65	Community based (86% rural)	Self-reported, Physician’s diagnosis	>13 (System wise)
6	Chakrabarty *et al*, 2010^26^	India	2007	495	>60	Community based (rural)	Self-reported, Physician’s diagnosis	16 (ICD-10 coding related diseases)
7	Bhojani *et al*, 2013^16^	India	June 2009–March 2010	44 514	>18	Community based (urban)	Self-reported	Any chronic conditions
8	Van Minh *et al*, 2008^20^	India Bangladesh	2005	India (N=2080) Bangladesh (N=8096)	25–65 years	Community based predominantly rural	Self-reported	7
9	Banjare and Pradhan 2014^28^	India	October 2011–February 2012	320	>60	Community based (rural)	Self-reported	20 listed
10	Pati *et al*, 2014^23^	India	2007–2010	10 978	>18	Secondary data based WHO-SAGE wave 1 (Community based; 75% Rural)	Self-reported	9
11	Arokiasamy *et al*, 2014^22^	India, China, Mexico, South Africa, Russia, Ghana	2007–10	11 230	>18	Secondary data based WHO-SAGE wave 1 (community based; 75% rural)	Self-reported	8
12	Vadrevu *et al*, 2015^29^	India	2009	815	≥40	Community based (rural)	Self-reported and symptoms based	6
13	Arokiasamy *et al*, 2015^21^	India	2010	1683	>45	Secondary data LASI LASI (72% rural)	Self-reported	7

ICD10, 10th revision of the International Statistical Classification of Diseases and Related Health Problems; LASI, Longitudinal Aging Study India; WHO-SAGE, World Health Organization- Study on global AGEing and adult health.

### Definition and estimation of multimorbidity

**‘**Multimorbidity’ was defined and used in six studies (table 2).^21–23^ ^25^ ^28^ ^29^ The remaining seven articles mentioned the presence of two or more chronic conditions without using the term ‘multimorbidity’. Twelve used a predefined list of chronic conditions ranging from 7 to 16, (see online supplementary appendix 3) and one adopted a free listing method.^16^ For identification of patients with chronic conditions, different approaches were used, namely, self-reports in five studies,^20^ ^21^ ^23^ ^27^ ^28^ self-reports and physician diagnosis in four studies,^24^ ^26^ ^30^ ^32^ and, in other studies, a combination of physician's diagnosis and laboratory investigations,^25^ and both self-reported and symptom based approaches were used.^22^ ^29^ International classification for disease coding was used in three studies and the remaining used arbitrary systems of coding.^26^ ^32^

**Table 2:** Characteristics of selected studies concerning prevalence of multimorbidity and risk factors

Author, year of publication	Country	Use of term multimorbidity in the study	Definition of multimorbidity	Results Prevalence	Risk factors	Consequences
Joshi 2003	India	No	Not described	83%*	Not described	No
Purty 2006	India	No	Not described	24.1%	Not described	No
Khanam 2011	Bangladesh	Yes	Two or more chronic medical conditions	53.8%	Women (OR 3.32; 1.88–5.86) High-income group (OR 1.93; 1.14–3.27)	No
Chakraborty 2004	India	No	Not described	54.4%		No
Swami 2002	India	No	Not described	69.9%	Urban Female	No
Chakrabarty 2010	India	No	Not described	53.7%	Not described	No
Bhojani 2013	India	No	Not described	4.5%	Not described	No
Van Minh 2008	India Bangladesh	No	Not described	India=5.86% Bangladesh=10.75%	Not described	No
Banjare 2014	India	Yes	Presence of two or more chronic diseases	56.5%	Age in years 65–70: (OR 2.33; 1.22–4.45) 70–75: (OR 4.91; 2.18–11.05) ≥75: (OR 4.65; 1.87–11.52) SES Fully dependent: (OR 5.21; 1.99–13.60) Partially dependent: (OR 3.02; 1.57–6.81) Chewing tobacco: (OR 2.82; 1.51–5.24)	No
Pati 2014	India	Yes	Presence of two or more chronic diseases	8.9%	Age in years 30–39: (OR 4.11; 2.18–7.74) 40–49: (OR 7.87; 4.25–14.59) 50–59: (OR 16.15; 8.83–29.54) 60–69: (OR 23.56; 13.08–42.44) >70: (OR 39.15; 20.72–73.98)	Increase healthcare utilisation and expenditure
Arokiasamy 2014	India	Yes	Simultaneous presence of two or more chronic diseases	20.8%	Age in years 50–59: (OR 3.12; 2.88–3.37) 60–69: (OR 5.24; 4.83–5.7) ≥70: (OR 7.53;6.9–8.26) Widow/er (OR 1.45; 1.26–1.64) Obese (OR 1.59; 1.48–1.71) High risk WHR (OR 1.25; 1.17–1.32) Inactive PA (OR 1.13; 1.07–1.19)	Poor self-rated health (SRH) Increased functional limitation Poor quality of life Depression
Vadrevu 2015	India	Yes	Presence of two or more chronic diseases	Not mentioned		Poor SRH
Arokiasamy 2015	India	Yes	Presence of two or more chronic diseases	9%	Age: 45–59 years (OR 1.5; 1.19–1.88) Primary education (OR 1.4; 1.02–1.91) High school and above (OR: 1.6; 1.07–2.4 Female (OR: 1.25; 0.97–1.61)	Poor ADL and SRH

ADL, activity of daily living; PA, physical activity; WHR, waist hip ratio.

Five studies had stated the objective of estimating the prevalence of multimorbidity.^21–23^ ^28^ ^29^ The rest were intended to identify multiple chronic conditions. The prevalence of multimorbidity varied from 4.5%^16^ to 83%.^32^ Among the population aged 60 years or over, the prevalence ranged from 24.1%^24^ to 83%,^32^ while for the remaining adult population it was from 4.5%^16^ to 20.8%.^21^ Prevalence of multimorbidity among studies adopting self-reported methodology ranged from 4.3%^20^ to 56.8%.^16^ Among studies from national representative samples, the prevalence varied from 4.3%^20^ to 8.9%.^23^ The only study using physician diagnosis and laboratory investigations reported multimorbidity prevalence of 53.8%.^25^ The prevalence varied from 24.1%^24^ to 83%^32^ among studies that used both a self-reported and clinical examination approach. All the studies had followed a simple count method adding up the number of chronic diseases.

### Patterns, correlates and consequences of multimorbidity

The leading chronic conditions reported were hypertension, arthritis, diabetes, cardiac problems and skin diseases (see online supplementary appendix 3). Apart from one,^28^ no other studies reported the pattern of diseases or commonly occurring disease clusters. The most frequently reported consequences were increase in healthcare utilisation,^23^ ^27^ ^30^ lowering of physical functioning,^21^ ^22^ disability,^32^ quality of life,^21^ healthcare expenditure^23^ and psychological distress.^32^ Only one study^21^ explored the morbidity burden or severity and HRQoL. Four studies^21–23^ ^28^ identified age to be strongly associated with multimorbidity. Two studies^22^ ^28^ considered risk factors such as tobacco use, obesity, waist hip ratio and physical activity, for prediction of multimorbidity. Three studies^21^ ^22^ ^29^ looked at the impact of multimorbidity on self-rated health. One study^23^ explored the effect of multimorbidity on healthcare utilisation and expenditure. Positive association between multimorbidity and depression was reported in two articles.^22^ ^32^

## Discussion

The present systematic review intended to summarise the scientific evidence accumulated in the past two decades pertaining to multimorbidity in South Asia. We identified only 13 studies, confined to two countries. Earlier reviews by Western authors also noted the limited representation of developing countries in multimorbidity research.^9^ South Asians have already been shown to be an inherently high-risk group for developing cardiometabolic and other chronic diseases, and thus multimorbidity may be significantly prevalent in these populations.^33^ Nevertheless, the scarcity of publications in our review demonstrates an obvious mismatch between the need for work versus work accomplished in this area.

Five studies had the primary objective of estimating multimorbidity, while for others, it was a secondary observation, which further reduces the strength of evidence on this topic. Interestingly, six studies have assessed the prevalence of two or more chronic conditions without citing the term ‘multimorbidity’, suggesting low familiarity of the researchers with this entity. Five were published in the year 2014–2015, indicating the recent growing interest in multimorbidity in this region. At the same time, it also suggests the continuing foothold of single disease and infectious conditions among South Asian health system researchers.

The wide variance in prevalence estimates observed in our review stems from the diversities in study methodologies. For instance, sample size estimation, age group of the study participants, and inclusion and exclusion criteria, differed considerably between studies, which makes comparability difficult. Similar heterogeneity was observed in a review documenting prevalence of comorbidities in Australia, where diverse methods and study settings were the contributing reasons.^34^ Another review on multimorbidity patterns also exhibited considerable methodological variability in terms of sample size, age and recruitment of study participants, data source and number of base line diseases.^35^ Four of the 13 studies used secondary data from national surveys.^20–23^ None of the reported studies had the intention or objective of looking at multimorbidity, per se.

Overall quality assessment revealed major lacunae in methodological aspects, which included ascertainment and case definition of multimorbidity, selection of source population, and inclusion and exclusion criteria. Even though some of these weaknesses were noted by the researcher in the limitations section, none of the studies tried to address bias. The wide heterogeneity observed due to non-uniformity in methodology and disease screening criteria makes comparability difficult and explains to some extent the large diversity observed. Owing to the inherent biases in the original studies’ estimation, quantification of the prevalence could not be assessed.

The majority of the authors did not describe the criteria for selection of chronic diseases. Where they did, the most common were those conditions with a high prevalence and/or clinical relevance. As the number and type of conditions included determines multimorbidity estimation, the reported prevalence in these studies may not be reflective of the real burden. Moreover, there was ambiguity in disease definitions, such as doubt over whether ischaemic heart disease and myocardial infarction should be considered separate entities. Thus, efforts should be first directed at preparing a panel of chronic diseases with standardised definitions of each condition. This would help in minimising the inter-study variations, reduce possible selection bias of specific chronic diseases, and result in more reliable and comparable estimation of multimorbidity. Further, none of the included studies were undertaken in a primary care setting, which constitutes an important knowledge gap and substantiates the earlier evidence of non-availability exploring multimorbidity in primary care settings in LMICs.^35^ In view of the integral role of primary care in the management of patients with long-term conditions,^6^ and primary care being the major healthcare provider for the population in this region,^36^ future studies should include these practices in exploring multimorbidity.

The study populations in most articles were aged 60 years or above, which might have introduced an element of age bias. One possible reason could be that most researchers have assumed multiple chronic conditions to be more akin to the geriatric population. Multimorbidity is not limited to old age alone, as it is significantly prevalent among the young population as well.^12^ Equating multimorbidity with ageing could underestimate its real magnitude. This has important implications, especially for South Asian countries, as the majority of this region's population is young and possesses the risk of escalation of burden of multiple chronic conditions in the future.

Many authors have emphasised the importance of examining the pattern of multimorbidity in addition to quantifying the conditions. Identification of high frequency clusters is important for developing specific treatment guidelines and better patient management. However, only one study in our review explored the clustering of diseases.^28^ The recent review on pattern of associative multimorbidity by Prados-Torres *et al*^35^ reflected similar findings with lack of published literature from LMICs.

The negative health consequences of multimorbidity have placed it in the forefront of healthcare and research, the most relevant sequelae being increased healthcare utilisation, decreasing HRQoL, impaired physical functioning, poor mental health and increased healthcare expenditure.^37^ ^38^ In our review, less than half the studies considered this aspect by assessing physical and mental functioning and healthcare utilisation. Few have looked at the impact of multimorbidity on HRQoL and self-reported health. In view of the informative role of outcome measurement in the design of interventions, future studies investigating the burden of multimorbidity in South Asia need to embrace this dimension.

Finally, the insufficient volume of published work gathered through our review is inadequate to provide an indication of the magnitude of multimorbidity in South Asian countries. This is both surprising and concerning since multimorbidity is a well-recognised priority in chronic disease research worldwide and no longer considered exotic. Increased research into clinical and epidemiological aspects of multimorbidity is essential to build up the scientific evidence in this geographic region. More importantly, the wide heterogeneity observed in the present review insinuates the need of greater methodological rigour while conducting these epidemiological studies.

### Study limitations

The major limitation of our systematic review is the difficulty in ensuring that all the relevant literature has been included. Since multimorbidity is not well indexed in literature databases we might have inadvertently omitted some studies. We tried to compensate for this by using an extended list of text words referring to the term multimorbidity as well as including any studies reporting two or more chronic conditions excluding comorbidity. Owing to the large heterogeneity among the studies, we could not perform quantitative synthesis of the prevalence estimates. An inherent limitation of any systematic review is the necessity to restrict a search period, which involves the exclusion of new studies after the end date. This might have resulted in omission of very recent studies.

## Conclusions and future research

Multimorbidity still remains an unexplored area of research in South Asia. Despite the growing prevalence of chronic diseases, the evidence base for multimorbidity and its consequences is extremely limited for this region. Since multimorbidity is a major challenge to primary care, prevalence studies in these settings are recommended. Further, relevant outcome measures such as healthcare utilisation, quality of life, activity of daily living and healthcare expenditure should be examined in unison with prevalence. Care should be taken to adopt a uniform operational definition of multimorbidity, and an iterative list of chronic conditions contextualised for individual countries should be developed while assessing multimorbidity.
